# Special Issue “Peptides for Health Benefits 2021”

**DOI:** 10.3390/ijms25042362

**Published:** 2024-02-17

**Authors:** Cristina Martínez-Villaluenga, Blanca Hernández-Ledesma

**Affiliations:** 1 Institute of Food Science, Technology and Nutrition (ICTAN-CSIC), Jose Antonio Novais 6, 28040 Madrid, Spain; c.m.villaluenga@csic.es; 2 Institute of Food Science Research (CIAL, CSIC-UAM, CEI UAM+CSIC), Nicolás Cabrera 9, 28049 Madrid, Spain

In recent years, non-communicable diseases (NCDs) have increased in prevalence in our society and have become a serious burden of disease worldwide. More than 50% of all deaths are attributed to them. Inflammation-related diseases such as stroke, cancer, ischemic heart disease, chronic kidney disease, and auto-immune and neurodegenerative conditions are some of the biggest threats and challenges to human health nowadays. Recent studies have suggested that the risk of developing chronic inflammation can be traced back to early development in the first stages of life, and its effects can persist throughout life, affecting adult health and mortality risk. The critical role that macrophages play in the inflammatory response to environmental triggers is well known, contributing to multiple disease processes, even chronic ones. Histatin peptides are recognized antimicrobial, wound-healing agents and contribute to environmental responses and immunomodulation. In the study of Lee et al. [1], the effect of histatin-1 on lipopolysaccharide (LPS)-induced macrophages RAW264.7 was investigated, elucidating the underlying mechanisms. The findings of this study demonstrated that this peptide could inhibit the production of inflammation mediators such as nitric oxide and cytokines and downregulate inflammatory signaling pathways.

The continuous increase in cancer incidence and mortality rates has made this disease one of the major public health problems worldwide. By 2040, cancer cases are expected to exceed 28 million, a 47% increase from 2020. These data imply an increase in the demand for therapies to reduce cancer mortality, but also for prevention alternatives to reduce the incidence of this disease. Lunasin is a natural soybean peptide whose proven properties have made it a promising preventive and therapeutic option against cancer. In the article by Alves de Souza et al. [2], the relationship between the structural characteristics of this peptide and its pharmacokinetics, safety, and bioactivity is summarized. In addition, these authors revised the underlying molecular mechanisms of action against cancer and discussed commercial lunasin-enriched products and potential combination therapeutical strategies. Peptaibols are a family of intrinsically stable helical 3D peptides synthetized by fungi as part of their defense mechanism against other microorganisms. It has been demonstrated that these peptides exert a potent anticancer activity through their capacity to alter the permeability of phospholipid membranes. However, their administration is limited by their natural hydrophobicity. In the study by Casagrande et al. [3], two water-soluble analogues of the peptaibol trichogin GA IV, termed K6-Lol and K6-NH2, were synthesized by a green methodology, and their activity was evaluated in cisplatin and doxorubicin-resistant ovarian cancer and Hodgkin lymphoma cell lines. The two compounds showed cytotoxic effects on both cancer cells, causing cell membrane permeabilization and exposure to phosphatidylserine. Their resistance to proteolysis and maintenance of the helical structure present these peptide analogues as promising new anticancer agents.

Based on the World Health Organization (WHO) data, cardiovascular diseases (CVD) are the leading cause of morbidity, disability, and mortality among NCDs, causing nearly 18 million deaths per year. These diseases include hypertension, coronary heart disease, cerebrovascular disease, and rheumatic heart disease. Considering that one third of all ischemic heart disease is due to elevated cholesterol levels, statins are one of the main therapeutic agents to treat hypercholesterolemia and prevent CVD. However, the side effects associated with prolonged use of these drugs limit their use and new natural strategies are in demand. Several studies have proposed that bioactive peptides, particularly those released from legume seed, represent a possible alternative to statin drugs. In the work of Silva et al. [4], an in silico approach was used to elucidate the mechanism underlying the binding of cowpea β-vignin-derived peptides and 3-hydroxy-3-methylglutaryl-coenzyme A (HMG-CoA) reductase. The results of this study guided the synthesis of three peptides, IAF, QGF, and QDF, capable of reducing cholesterol synthesis through a mechanism similar to that of statins. By using tandem mass spectrometry and de novo sequencing, Lopez-Huertas and Alcaide-Hidalgo [5] identified nineteen sequences as endogenous peptides present in virgin olive oil. BIOPEP software was used to predict the biological activities of these peptides and estimate their behavior under simulated gastrointestinal digestion. Peptides VCGEAFGKA, NALLCSNS, CPANGFY, CCYSVY, and DCHYFL were found to exert potent angiotensin-converting enzyme (ACE) inhibitory and antioxidant properties. 

It is well recognized that the development and progression of neurodegenerative diseases are accelerated due to oxidative stress and inflammation, which result in the impairment of mitochondrial function, cellular damage, and dysfunction of DNA repair systems. Prolonging life span in developed countries contributes to an increase in the incidence ratio of chronic age-related neurodegenerative disorders, such as Parkinson’s disease (PD), Alzheimer’s disease (AD), or numerous forms of age-related dementias. AD is a devastating neurodegenerative disease characterized by progressive neuron loss in memory-related brain structures. New therapeutic strategies have emerged, such as the exogenous administration of neurotrophic factors (e.g., NGF and BDNF) that are deficient or dysregulated in patients with AD. Gascon et al. [6] reviewed growth factors and their derived peptides as potential treatments for AD. Their review describes (1) the physiological functions of growth factors in the brain, their neuronal signaling pathways, and alterations in AD; (2) the strategies to develop peptides derived from growth factors and their capacity to mimic the role of native proteins; and (3) new advancements in and the potential use of these molecules as therapeutic treatments for AD, as well as their limitations. Osteopontin is a multifunctional glycoprotein expressed in numerous cell types which play important roles by interacting with multiple receptors, including integrins and CD44 variants. This protein has been found to exert neuroprotective effects against several diseases such as PD, multiple sclerosis, and subarachnoid or intracerebral hemorrhage. An icosamer of this protein, containing RGD and SLAY motifs, has also been demonstrated to exert neuroprotective effects in an animal model of transient focal ischemia mediated by anti-inflammatory, pro-angiogenic, and immunostimulating functions. In the study by Davaanyam et al. [7], the icosamer was truncated and the neuroprotective efficacy of the resulting peptides containing RGD and/or SLAY motifs was examined in a rat middle cerebral artery occlusion model after their intranasal administration. The most potent effect was observed for the RGD-7-amino acid peptide that was able to suppress pro-inflammatory M1 markers and augment anti-inflammatory M2 polarization of microglia. Pituitary adenylate cyclase-activating polypeptide (PACAP) is a neuropeptide widely distributed in the central nervous system (CNS) and many peripheral organs. In the gastrointestinal (GI) tract, PACAP plays an important regulatory function. PACAP stimulates the secretion of digestive juices and hormone release, and regulates smooth muscle contraction, local blood flow and cell migration and proliferation. Additionally, there are many reports confirming the involvement of PACAP in pathological processes within the GI tract, including inflammatory states, neuronal injury, diabetes, intoxication, and neoplastic processes. Karpiesiuk et al. [8] reviewed the distribution and pleiotropic action of PACAP in the control of gastrointestinal tract function and its cytoprotective effect in the course of gastrointestinal tract disorders. In another article, Palus et al. [9] elucidated the impact of prolonged hyperglycemic conditions on a population of PACAP-like immunoreactive neurons in selected parts of the porcine gastrointestinal tract. The results suggested that PACAP is involved in regulatory processes of the gastrointestinal tract function in the course of diabetes. 

Parathyroid hormone-related protein (PTHrP) C-terminal peptides regulate the metabolism of bone cells. PHTrP (osteostatin) promotes bone repair in animal models with bone defects, prevents bone erosion in inflammatory arthritis, and inhibits bone resorption. Ibáñez et al. [10] determined the effects of osteostatin on human osteoclast differentiation and function. These authors demonstrated that osteostatin controls human osteoclast differentiation in vitro through the downregulation of nuclear factors of activated T cells, cytoplasmic 1. In addition, antiresorptive effects of osteostatin were dependent on the inhibition of osteoclastogenesis. 

Mitochondria play central roles in maintaining cellular metabolic homeostasis, contribute to cell survival and cell death, and generate most of the cell’s energy. Pink1 is a serine/threonine kinase which regulates mitochondrial function, yet many molecular mechanisms underlying Pink1 activity in mitochondrial homeostasis and cell fate remain unknown. Ben-Uliel et al. [11] rationally designed a linear peptide that targets Pink1 and developed molecular probes with drug-like properties to further characterize Pink1. Overall, this study offers a new approach to converting a non-permeable linear peptide into a research tool possessing important properties for therapeutics.

The naturally occurring dipeptide carnosine (β-alanyl-l-histidine) has beneficial effects in different diseases, improves exercise performance, and exerts antiaging effects. Oppermann et al. [12] investigated the uptake and intracellular amounts of carnosine and its effect on ATP production in human erythrocytes and on their response to oxidative stress. The results from this study demonstrated that erythrocytes could take up carnosine and, most importantly, thereby prevent its degradation by human serum carnosinase.

Human Ezrin peptides (HEPs) are cell membrane-associated proteins with multiple binding sites to cell surface receptors, intracellular kinases, and actin that form protein complexes implicated in cell signaling, shape, and motility. Among them, HEP1, whose sequence is TEKKRRETVEREKE, was registered for human use in Russia in 2001 under the trademark Gepon. Since its identification, multiple studies have demonstrated the benefits that this peptide can exert against opportunistic infections in HIV-infected patients such as mucosal candidiasis, herpes zoster outbreaks, and infection-induced chronic diarrhea. The beneficial actions attributed to this peptide and its derivative GEKKRRETVEREGG are revised in the article by Holms and Ataullakhanov [13]. In addition, this review also summarizes the existing evidence on the effects of these HEPs as agents for the treatment of acute viral respiratory disease with inflammatory complications, adjuvants to immune therapies, and potential drugs in the treatment of COVID-19.

Decades of study have delineated the biological role of the nociceptin/orphanin FQ system, demonstrating its involvement in significant physiological processes such as pain, learning and memory, anxiety, depression, feeding, and drug and alcohol dependence. Ubaldi et al. [14] reviewed the role of this peptidergic system in the modulation of stress and stress-associated psychiatric disorders, in particular drug addiction, mood, anxiety, and food-related associated-disorders. The current literature suggests that nociceptin opioid-like receptor antagonists can be useful to treat depression and feeding-related diseases, such as obesity and binge eating disorder, whereas the activation of nociceptin opioid-like receptor agonists could be a promising tool for anxiety. The review by Kaczyńska and Wojciechowski [15] summarizes the most recent evidence on the modulatory effects of various endogenous and exogenous peptides on different opioid responses, with special emphasis on the cardiovascular and respiratory level. In this review, the available findings on neuropeptide FF, cholecystokinin, melanocyte inhibitory factor, nociceptin/orphanin, ghrelin, oxytocin, endothelin, and venom peptides are described. The review by Redkiewicz et al. [16] discusses in depth the data available after 40 years of research on the properties of biphalin, a dimeric analogue of enkephalin with a high affinity for opioid receptors. Its summarized analgesic, neuropathic, antipain, antiviral, antiproliferative, anti-inflammatory, and neuroprotective effects make this peptide a promising therapy for many opioid system-dependent pathological diseases.

In the fast-developing field of tissue engineering, there is a constant demand for new materials to be used as scaffolds for cell seeding which can better mimic a natural extracellular matrix as well as control cell behavior. Golunova et al. [17] focused on bone sialoprotein-derived peptide (TYRAY) conjugation to the molecule of alginate using four different approaches to provide biofunctionality to the alginate structure for cell adhesion. The combination of the alginate amidation with the use of subsequent Cu-catalyzed azide–alkyne cycloaddition led to efficient peptide conjugation and adhesion of human embryonic stem cells.
